# Targeting the Oxytocinergic System: A Possible Pharmacological Strategy for the Treatment of Inflammation Occurring in Different Chronic Diseases

**DOI:** 10.3390/ijms221910250

**Published:** 2021-09-23

**Authors:** Marzia Friuli, Barbara Eramo, Marta Valenza, Caterina Scuderi, Gustavo Provensi, Adele Romano

**Affiliations:** 1 Department of Physiology and Pharmacology “V. Erspamer”, Sapienza University of Rome, 00185 Rome, Italy; marzia.friuli@uniroma1.it (M.F.); barbara.eramo@uniroma1.it (B.E.); marta.valenza@uniroma1.it (M.V.); caterina.scuderi@uniroma1.it (C.S.); 2 Department of Neuroscience, Psychology, Drug Research and Child Health (NEUROFARBA), Section of Pharmacology of Toxicology, University of Florence, 50139 Florence, Italy; gustavo.provensi@unifi.it

**Keywords:** oxytocin, inflammation, glia, chronic diseases, therapeutic strategy

## Abstract

Unresolved inflammation represents a central feature of different human pathologies including neuropsychiatric, cardiovascular, and metabolic diseases. The epidemiologic relevance of such disorders justifies the increasing interest in further understanding the mechanisms underpinning the inflammatory process occurring in such chronic diseases to provide potential novel pharmacological approaches. The most common and effective therapies for controlling inflammation are glucocorticoids; however, a variety of other molecules have been demonstrated to have an anti-inflammatory potential, including neuropeptides. In recent years, the oxytocinergic system has seen an explosion of scientific studies, demonstrating its potential to contribute to a variety of physiological processes including inflammation. Therefore, the aim of the present review was to understand the role of oxytocin in the modulation of inflammation occurring in different chronic diseases. The criterion we used to select the diseases was based on the emerging literature showing a putative involvement of the oxytocinergic system in inflammatory processes in a variety of pathologies including neurological, gastrointestinal and cardiovascular disorders, diabetes and obesity. The evidence reviewed here supports a beneficial role of oxytocin in the control of both peripheral and central inflammatory response happening in the aforementioned pathologies. Although future studies are necessary to elucidate the mechanistic details underlying such regulation, this review supports the idea that the modulation of the endogenous oxytocinergic system might represent a new potential pharmacological approach for the treatment of inflammation.

## 1. Introduction

### 1.1. Inflammation

A physiologic inflammatory response leads to the upregulation of inflammatory activity, which is temporally confined when a threat is present and which ends when the threat has been eliminated [1,2]. However, in the presence of some genetic, biological, psychological, and environmental circumstances, the resolution of the acute inflammatory process is abolished, thus allowing the occurrence of a state of a chronic low-grade systemic inflammation, characterized by the stimulation of immune components different from those activated during the acute immune response [1,3]. However, both in the case of chronic and acute inflammation, resolution requires the activation of endogenous programs with a shift from the stimulation of pro-inflammatory products towards pro-resolving molecules [4]. The initiation and resolution of inflammation is mediated and finely controlled by different types of mediators [5]. Indeed, in the initial stage of systemic inflammation, resident immune cells, such as macrophages, mast cells, and dendritic cells, are activated at the site of infection/injury and produce soluble mediators, including chemokines and cytokines [6,7]. Subsequently, plasma proteins and leukocytes (mainly neutrophils), normally restricted to the blood vessels, gain access to the extravascular tissues at the site of infection/injury. The activated endothelium of the blood vessels allows selective extravasation of neutrophils that react, releasing the toxic contents of their granules to kill the invading pathogens or digest the harmful agent that triggered the inflammatory process [8].

One of the most interesting discoveries of the past years has been that inflammatory processes are not only involved in a few selected disorders, but in a plethora of mental and physical pathologies that are responsible for mortality worldwide [9,10,11,12,13,14].

Therefore, signs of stubborn, unresolved inflammation represent features of different human chronic pathologies including stroke, cardiovascular disease, cancer, and diabetes [15]. The epidemiologic relevance of such disorders justifies the increasing interest in further studying the inflammatory machinery to understand the molecular and cellular mechanisms involved in the inflammatory response, to provide potential novel pharmacological targets for the treatment of inflammation occurring in such diseases [9].

In recent years, both in vitro and in vivo studies have demonstrated an anti-inflammatory potential of a variety of molecules different from the classical glucocorticoids. Among such molecules, particular interest has been attributed to neuropeptides. Neuropeptides are small aminoacidic molecules produced mainly, although not exclusively, by cells of the central nervous system (CNS). Usually, they are capable of regulating neuronal activity and affecting a great variety of central and systemic functions, such as food and water intake, thermoregulation, circadian rhythms, and reproductive behavior [16].

While some neuropeptides including substance P and calcitonin gene-related peptide have long been recognized to be mediators of inflammation and tissue repair [17], accumulating evidence suggests that also the neuropeptide oxytocin participates in such processes.

### 1.2. The Oxytocinergic System

In recent years, the oxytocinergic system has seen an explosion of scientific studies, showing that oxytocin modulates a wide range of neurotransmitter and hormones’ activities as well as physiologic functions [18], thus producing a more comprehensive picture of the oxytocinergic signaling and the pathways that regulate its release and degradation.

Oxytocin is a peptide hormone mainly produced by two hypothalamic nuclei, supraoptic and paraventricular ones. Even though the magnocellular neurons of both paraventricular and supraoptic nuclei are the major ones responsible for oxytocin production, a contribution is also made by the smallest parvocellular neurons [19,20].

Following stimuli such as childbirth and breastfeeding, oxytocin exerts autocrine and paracrine actions at the level of the hypothalamus through a somato-dendritic release [19]. Interestingly, an intrinsic regulation of the oxytocinergic system was examined by Honda and colleagues who demonstrated the presence of excitatory synaptic projections between supraoptic and paraventricular oxytocin neurons that might probably be responsible for the synchronization of burst discharges of oxytocin cells within the hypothalamus [21].

Oxytocin is released in the bloodstream through the neurohypophysis, which receives axonal projections from magnocellular neurons (a neuroendocrine component of the oxytocinergic system) [19,20].

On the other hand, parvocellular oxytocin neurons project to distinct areas of the brainstem and spinal cord and, relatively recently, it has been suggested that they also project to magnocellular oxytocin neurons of the supraoptic nucleus, to modulate the release of oxytocin into the blood [20,22,23].

The oxytocin receptor is part of the superfamily of G protein-coupled receptors. In particular, this receptor has been traditionally identified as being associated with a Gq/11α [24], although recent studies have shown that the oxytocin receptor can also be coupled to inhibitory (Gi) or stimulatory (Gs) G protein, according to the location and physiological state of the animal [19,25].

The expression of the oxytocin receptor was detected in a variety of brain areas where it participates in the regulation of different functions, e.g., amygdala (social behavior and fear), hippocampus (spatial memory and neurogenesis), prefrontal cortex (maternal, socio-sexual, and anxiety behavior), hypothalamus (homeostatic feeding), periaqueductal gray (anxiety-related behaviors), olfactory bulb (social behavior), striatum, ventral tegmental area, and nucleus accumbens (non-homeostatic food intake and reward) [19,26,27,28,29,30]. Furthermore, it has been proposed that oxytocin released from the axonal terminals of parvocellular neurons projecting to the brainstem and spinal cord contributes to the modulation of cardiovascular functions, breathing, and nociception [31,32,33].

Outside the CNS, the expression of oxytocin receptor has been identified in different districts, including cardiomyocytes (regulation of ionotropic and chronotropic negative cardiac regulation) [34], adipocytes (stimulation of glucose-oxidation and lipogenesis) [24], and nociceptive ganglion neurons of the dorsal root (modulation of nociception targeting C-fibers) [35]. Oxytocin receptors have also been found in osteoblasts and osteoclasts, where oxytocin exerts an anabolic action [36], in the gastrointestinal tract, specifically in enteric neurons and enterocytes where it has been seen to modulate gastrointestinal motility and permeability [37], and in fibroblasts and in keratinocytes of the skin, with a role in the regulation of skin processes such as proliferation, inflammation, and oxidative stress responses [38].

The majority of the studies conducted on the oxytocinergic system focused on its capability to regulate maternal, social, and eating behavior [27,39], with very consistent results highlighting its potential to represent the keystone for the development of a novel pharmacological strategy to control disorders in such contexts; interestingly, new evidence highlights the implication of the oxytocinergic system also in the control of inflammatory processes [40], thus adding novel important properties to such neuropeptides [41].

Indeed, oxytocin has been shown to decrease the levels of pro-inflammatory cytokines induced by bacterial endotoxin lipopolysaccharide (LPS) [42] and to reduce edema in an animal model of inflammation [43]; moreover, a regulatory role in inflammatory responses, such as wound healing, has also been proposed [44]. Interestingly, oxytocin affects the modulation of cells’ proliferation, reactive oxygen species’ (ROS) formation, glutathione (GSH) content, and cytokines release in dermal fibroblasts and keratinocytes in patients with atopic dermatitis [38]. Finally, an interplay between oxytocin and glia has also been suggested (please see the next paragraph for more details) [45].

Based on these previous reports, in the present review we aimed to understand the role of oxytocin in the modulation of inflammation occurring in different chronic diseases, to open the way to novel therapeutics targeting the oxytocinergic system to ameliorate the inflammatory process associated with such diseases. The criterion that we used to select the diseases was based on the emerging literature showing a putative involvement of the oxytocinergic system in inflammatory processes occurring in different pathologies including neurological, gastrointestinal and cardiovascular disorders, diabetes and obesity. The data collected in the present work derive from animal data and are preclinical data; no clinical data are available at the moment. Indeed, the theme faced in the present review is pretty unexplored by the scientific community and, to the best of our knowledge, no review has been published collecting the evidence regarding the cross talk between the oxytocinergic system and the inflammatory machinery in such chronic diseases.

## 2. Oxytocin–Glia Communication

Glial cells are a heterogeneous class of cells usually classified into macroglia of ectodermal, neuroepithelial origin, and microglia of mesodermal, myeloid origin. The macroglia includes astrocytes, oligodendrocytes, and NG-2 glia (also known as oligodendroglial precursor cells or synantocytes) [46]. Despite their extreme heterogeneity, the fundamental unifying function of all types of glial cells is the defense of the CNS. They actively participate in all the processes aimed at restoring homeostasis and have a leading role in the control of neuroinflammation [47]. Indeed, whenever a brain injury occurs, glial cells become reactive, starting the so-called reactive astrogliosis, which is a constitutively, graded, and evolutionarily conserved defensive reaction [48]. However, reactivity causes many other modifications in glial cells that, if not stopped, can induce synaptic dysfunction, homeostatic imbalance, neurovascular unit dysfunction, loss of three-dimensional network, and blood–brain barrier alteration [49]. Interestingly, neural progenitor cells, microglia, and astrocytes express oxytocin receptors [50,51,52,53] and several in vitro and in vivo studies suggest that oxytocin treatment could exert effects in all glial cell types, either directly or indirectly. However, the mechanisms of action, for the most part, need to be clarified yet. For instance, it has been shown that cortical hippocampal primary astroglial cultures proliferate in the presence of oxytocin [54].

Additionally, oxytocin in vitro treatment stimulates the proliferation of cells of a human glioblastoma cell line through activation of the mitogen-activated protein kinase extracellular signal-regulated kinase (MAPK-ERK) 1/2 signaling pathway, which was blocked by the genetic knockdown of the oxytocin receptor [55]. Systemic administration of oxytocin to rat pups for 5 days (post-natal days 2–6), in the absence of any other challenge, caused an increase in gene and protein expression of the astrocytic marker glial fibrillary acidic protein (GFAP), with no change in the microglial marker of activation cluster of differentiation (CD) 68, and a small decrease in 2′,3′-Cyclic-nucleotide 3′-phosphodiesterase (CNPase), a marker specific for oligodendrocytes [56]. Similarly, 2 days of oxytocin administration (post-natal days, 2–3) to rat neonates induced a decrease in the mRNA levels of CNPase, with no change in the expression of CD68. In contrast to the previous study, GFAP gene expression was found to be reduced [57]. Interestingly, Mittaud and collaborators noticed that astrocytes express the oxytocin receptor when cultured alone, but they do it rarely when co-cultured with neurons, suggesting that neurons could exert a repressive effect on astrocytes. Both oxytocin receptor binding and oxytocin receptor mRNA levels were upregulated in rat hypothalamic astrocytes cultured adding neuron-conditioned medium as well as in co-culture of neurons and astrocytes without physical contact, whereas they were differently regulated in cultures where physical contact between astrocytes and neurons occurred. The authors suggested that some released factors, including the transforming growth factor (TGF) β, could be responsible for this cross talk between different cells [58]. Communication between oxytocinergic neurons and glial cells have several functional consequences, including regulation of extracellular ionic homeostasis, glutamate clearance, and, consequently, neuronal excitability [59]. Both in vivo and in vitro evidence shows that oxytocin triggers morphological changes in those astrocytes found juxtaposed to hypothalamic supraoptic neurons [45,60]. Indeed, glial morphological remodeling in the stimulated neurohypophysis leads to increased levels of extracellular potassium, which enhances neurohormone release, while a greater neurovascular contact zone may facilitate the diffusion of oxytocin into the circulation [59]. In hippocampal slices, the aforementioned reduction of astrocytic coverage of oxytocinergic neurons was rapid, but transient, and mediated by the interaction with the oxytocin receptor. Moreover, it could be potentiated by estrogen co-treatment [60]. In an ex vivo study, oxytocin evoked directly GFAP reduction in hypothalamic slices. Application of a gliotoxin to hypothalamic slices, by impairing astrocytes’ activities, reduced the excitatory postsynaptic currents of oxytocinergic neurons measured by patch-clamp recordings. The same authors also revealed that both GFAP protein levels and the association of GFAP with the astrocytic water channel aquaporin (AQP) 4 significantly decreased in the supraoptic nucleus during suckling. Both partially recovered after milk ejection reflex [53]. These findings suggest that the activity of the astrocytic AQP4 plays a crucial role in facilitating the astrocytic morphological changes, and in particular during lactation [61,62]. All this evidence demonstrates that the physiological activity of oxytocinergic neurons depends directly on astrocytic plasticity [45,53,61] and that lactation failure is associated with aberrant GFAP filament modifications during breastfeeding [61]. Oxytocin exerts anti-neuroinflammatory effects by limiting oxidative stress and pro-inflammatory cascades [40,63]. Additionally, oxytocin receptor knockdown in hippocampal primary astrocytes was found to activate the Nod-like receptor protein 33 inflammasome [64].

Regarding oxytocin effect in microglial cells, in vitro oxytocin treatment of LPS-challenged microglial cell line suppresses the elevation in gene expression of proinflammatory mediators, such as Tumor Necrosis Factor (TNF) α, interleukin (IL) 1β, cyclooxygenase (COX) 2, and inducible nitric oxide synthase (iNOS), together with LPS-induced phosphorylation of p38-MAPK and ERK [42]. It also reduced the elevation in intracellular Ca^2+^, which is essential for proliferation, migration, change in morphology, and activation of cell death program, as well as for the release of trophic and signaling factors, including proinflammatory cytokines [65,66]. Oxytocin in vitro treatment of LPS-exposed microglia attenuated also the LPS-induced expression of the major histocompatibility complex class II in a dose-dependent manner [67]. In vivo, intranasal oxytocin administration in LPS-challenged mice reduced their cortical neuroinflammatory parameters, including both the expression of TNF-α and IL-1β, as well as LPS-increased ionized calcium-binding adapter molecule (Iba) 1 immunostaining [42]. The inhibition of proinflammatory markers by oxytocin seems to be mediated through its receptor, which has been found to be upregulated following in vitro exposure to LPS [42]. Another study reported the involvement of the endoplasmic reticulum stress-related signaling, specifically the eukaryotic initiation factor 2/activating transcription factor 4 pathway, in the mechanism used by oxytocin to reduce the production of proinflammatory cytokines by LPS-challenged primary microglia [68]. Regardless of the mechanism adopted, together all this evidence suggests that oxytocin could attenuate microglia reactivity. This notion is supported also by in vivo data reporting a rise in circulating oxytocin 6 h after cecal ligation and puncture in rats, a surgical method to induce sepsis. This increase in oxytocin release was prevented by pretreatment with minocycline, a tetracycline that acts also as an inhibitor of microglia reactivity [69]. Further, minocycline treatment of mice in which the oxytocin receptor gene is fully ablated (OXYR-KO) restored normal levels of the postsynaptic density protein (PSD) 95, as well as a behavioral measure of dam-pup communication, suggesting that the inhibition of microglia reactivity could ameliorate the synaptic loss seen in these mice [70]. In two different in vivo models of perinatal inflammation, administration of a selective agonist of the oxytocin receptor, carbetocin, induced a significant reduction of microglial morphological changes related to reactivity, as well as preventing the up-regulation of proinflammatory markers (including IL-6, IL-1β, TNF-α, and iNOS) and ameliorating the myelinization, which was seen both at histological and imaging examinations, ultimately improving performance at behavioral testing [71]. Therefore, the agonism to the oxytocin receptor, mediated by either the endogenous or an exogenous molecule, could exert anti-neuroinflammatory properties that could be neuroprotective in the developing brain that has been injured perinatally [72,73,74]. However, a report suggests that perinatal exposure to exogenous oxytocin could have neurodevelopmental consequences for the fetus; despite that synthetic oxytocin does not alter in vitro proliferation of neural progenitor cells, it promotes their spontaneous differentiation preferentially into neurons more than astrocytes and oligodendrocytes [51]. Additionally, oligodendrocytes were found to be more vulnerable to the brain damage caused by oxytocin-induced labor in term-pregnant dams than other brain cells, such as neurons, astrocytes, and microglia. This oligodendrocyte-specific impairment during the perinatal period was then found in some way counterbalanced by increased numbers of oligodendrocyte precursor cells a few days after birth. Moreover, the fetal brain expression of both oxytocin and its receptor seemed not to be altered by the exogenous oxytocin administered to the mothers during labor [75]. Oxytocin administration seems to exert effects also peripherally, by counteracting some alterations in enteric glial cells, turned into reactive cells in pups following maternal separations. Neonatal maternal separation causes visceral hypersensitivity together with reactivity of enteric glial cells; it significantly increases the expression of the oxytocin receptor and hyperactivity of the Toll-like receptor (TLR) 4 signaling in the colon. Oxytocin treatment reduced behavioral measures of visceral hypersensitivity, which was reversed by the administration of atosiban, an oxytocin receptor antagonist. Finally, in vivo oxytocin treatment suppressed the maternal separation-induced TLR-4 signaling as well as the release of the downstream proinflammatory effectors (myeloid differentiation primary response 88, nuclear factor, NF κB, IL-1β, and TNF-α), reducing also enteric GFAP expression [76].

## 3. Oxytocin Signaling and Inflammation Occurring in Chronic Diseases

### 3.1. Neurological Diseases

Although the monoaminergic hypothesis has dominated for the last decades the understanding of the pathophysiology of depression by offering a variety of antidepressant drugs [77], recently the inflammatory hypothesis of depression has been highlighted. 

Indeed, several lines of evidence suggest that the inflammatory process is one of the main contributing factors to the pathogenesis of depression [78,79,80]. Therefore, it has been shown that depressive-like behavior in different animal models is associated with an increase of pro-inflammatory cytokines and an increased expression of TLR, lipid peroxidation, and anti-inflammatory cytokine IL-10. Interestingly these alterations are restored by the administration of antidepressant drugs [81,82]. Furthermore, the mechanism of action of antidepressant drugs, such as selective serotonin and norepinephrine re-uptake inhibitors, has also been expanded in the direction of their anti-inflammatory action, which is supposed to include reduction of both peripheral and central levels of pro-inflammatory cytokines and the modulation of their synthesis mechanisms (such as NF-κB pathway, inflammasome activation, TLR activation) [83].

The oxytocinergic system has received a great deal of attention for its antidepressant properties [84], and some studies reported that the antidepressant beneficial effects of oxytocin are partly due to the attenuation of neuroinflammation.

It is very well known that animals separated from their mothers at birth develop depressive-like behavior [85]. Amini-Khoei and colleagues demonstrated that such behavior is associated with impaired mitochondrial functioning and immune-inflammatory response at the level of the hippocampus in male mice separated from their mothers at birth (post-natal days 2 to 14). Interestingly, the intracerebroventricular administration of oxytocin in such animals attenuated the depressive-like behaviors induced by maternal separation, as resulted from behavioral analyses using open-field and sucrose-preference tests. The beneficial effect of oxytocin is paralleled to an improvement of mitochondrial function (measured in terms of ROS production and GSH content in the hippocampus) and the decrease in the hippocampal expression of immune-inflammatory genes (such as TNF-α, IL-1β, and TLR-4) [63].

Depression is also a possible feature of cancer patients who have received chemotherapy [86,87]. Interestingly, Walker et al. hypothesized that altered exploratory and depressive-like behaviors resulting from chemotherapy were due to an increase in cytokine production, and they wanted to investigate whether social enrichment might mitigate such an increase. Therefore, they used female Balb/C mice to which a chemotherapeutic cocktail made of doxorubicin and cyclophosphamide was administrated and, after that, mice were isolated or group-housed. Chemotherapy-treated, socially isolated mice showed an increase in depressive-like behavior, measured by a forced swim test and open-field test, associated with increased IL-6 levels in the hippocampus. In group-housed mice with the same treatment this increase was attenuated, suggesting that social enrichment could have a protective effect on the development of depressive-like behavior in cancer subjects. Interestingly, the intracerebroventricular administration of oxytocin in chemotherapy-treated and isolated mice was able not only to attenuate the levels of IL-6 in the hippocampus, but also to dampen the depressive-like phenotype. Moreover, the administration of an oxytocin antagonist in both not-isolated mice and those subjected to chemotherapy led to the development of a phenotype comparable to that of isolated mice, thus suggesting that social enrichment dampens chemotherapy-induced increased inflammation and depressive-like behavior by recruiting the oxytocinergic system [88].

Finally, Qui et al. [89] investigated the effect of oxytocin in adolescent rats, born from a rat model of de novo post-partum depression, treated with corticosterone in association or not with the antidepressant fluoxetine. In the adolescent rat, corticosterone caused an increase of hippocampal neuroinflammation (measured by analyzing the tissue levels of IFN- γ, IL-1β, IL-6, IL-13, IL-10, IL-5, TNF-α, and IL-4 and chemokine C-X-C motif ligand 1 tissue levels) and a decrease in social investigation and impaired neurogenesis. Importantly, oxytocin treatment decreased neuroinflammation (limited to IL-6:IL-10 ratio), increased social investigation, and stimulated neurogenesis.

Investigations on the oxytocinergic system have also been shown that this system might be considered as a potential pharmacological candidate for the treatment of the social dysfunctions occurring in autism spectrum disorder (ASD) [90,91,92,93].

Although the bases of ASD are not yet well known, several lines of both preclinical and clinical research support the hypothesis that the inflammatory machinery may be involved in ASD [94,95,96]. Indeed, an increase of both peripheral and central levels of TNF-α, IL-1β, and IL-6 and activation of microglia have been found in animal models of ASD [97]. In addition, a clinical study conducted by Vargas and collaborators demonstrated that ASD patients show increased brain levels of pro-inflammatory cytokines, such as TNF-α, IFN-γ, and IL-6, and of the chemokine monocyte chemoattractant protein 1, particularly at the level of the cerebellum and prefrontal cortex [98]. Finally, also, El-Ansary and colleagues revealed that biochemical parameters related to inflammation, such as heat-shock-protein 70, TGF-β, caspase 7, and IFN-γ, are increased in blood samples obtained from autistic male subjects aged from 3 to 16 [99].

The role of oxytocin in ASD has also been investigated by focusing the research on its impact on inflammation. For instance, a negative correlation has been found between plasma levels of oxytocin and IFN-γ-induced protein-16 (a protein involved in various physiological processes, which, if released extracellularly, is able to act as a molecular pattern associated with damage resulting in inflammation [100]) in autistic male patients [101].

Wang et al. studied oxytocin treatment in a model of ASD obtained by administering valproate to pregnant dams to induce ASD in the offspring. The pups were then divided into three groups: control, autism, and autism + oxytocin treatment. Behavioral tests (open-field test, tail suspension test, Marble burying test, and three-chamber social interaction test) highlighted the development of an anxious, depressive-like phenotype and altered social behavior, which was improved by intranasal administration of oxytocin. Moreover, the authors found an increase in the levels of pro-inflammatory cytokines such as IL-6, TNF-α, and IL-1β as well as an increase in malondialdehyde and ROS within the hippocampus and amygdala. Interestingly, oxytocin treatment was also able to significantly reduce markers of inflammation and oxidative stress and restore antioxidant enzyme activity and GSH levels. Moreover, Iba1 immuno-positive cells were increased in the amygdala and cortex of the offspring compared with pups born by a control dam, and the acute intranasal administration of oxytocin attenuated this rise [102].

These results suggest that oxytocin treatment improves autism symptoms in a mouse model of ASD, by ameliorating oxidative stress and inflammation [102,103]. It is important to report that, in different brain regions correlated with ASD, oxytocin receptor-KO mice showed reduced expression of the PSD95 together with a two-fold increase in Iba1; their microglial cells are enlarged and present more branches compared with wild-type animals [70]. Indeed, the involvement of glial cells’ alterations in ASD has been recognized [96,104,105,106,107], but the mechanisms linking the oxytocin receptor function and microglia reactivity need still to be fully elucidated. Clarifying the correlation between neuroendocrine factors and microglia may contribute to shedding light upon the pathophysiology of many disorders including ASD. The evidence collected in this paragraph is summarized in Table 1.

### 3.2. Inflammatory Pain

Among the physiological functions governed by the oxytocinergic system, it has well-recognized capability to modulate nociception and pain response [35,129,130,131]. On this line, Nersesyan et al. aimed to investigate the mechanism by which oxytocin induces analgesia, highlighting the role of the type 1 vanilloid receptor (TRPV1) [132]. TRPV1 is a polymodal ion receptor and is the natural ligand of capsaicin. Despite its functions as a mediator of painful perception, its repetitive activation in sensory neurons determines a state of refractoriness of the channel, with consequent analgesic effect [133]. Oxytocin directly activates TRPV1, thus suggesting that the analgesic action of such peptide might be mediated by this receptor [132]. However, despite its analgesic properties, oxytocin is currently not used for the treatment of pain. Interestingly, in recent years, increasing emphasis has been placed on the role of oxytocin in the modulation of inflammatory pain [134]. Indeed, Eliava et al. [108] identified a subpopulation of oxytocin parvocellular neurons in the paraventricular nucleus that projects to the deep layers of the spinal cord. To investigate the functional importance of these parvocellular neurons the authors stimulated them, by an optogenetic approach, in a rat model of painful peripheral inflammatory sensitization caused by unilateral intraplantar injection of complete Freund adjuvant (CFA); the results showed that such stimulation is able to inhibit spinal pain processing by attenuating the nociceptive transmission. This effect was strictly linked to the oxytocinergic transmission since it was completely blocked by the intraperitoneal administration of an oxytocin receptor antagonist [108]. These findings suggest a role of these newly identified oxytocin parvocellular neurons in mitigating the hypersensitivity resulting from inflammatory pain.

Additionally, Hilfiger et al. reported the effects of a selective oxytocin receptor agonist on inflammatory pain, in a rat model of pain in which male rats were given subcutaneous CFA injection. Interestingly, the intraperitoneal administration of the selective oxytocin receptor agonist (LIT-001) raised both mechanical and thermal pain thresholds from 1 to 5 hours after its administration, with a maximum effect observed at 3 hours. The crucial involvement of the oxytocin receptor in such a context was confirmed by the administration of a selective oxytocin receptor antagonist, which completely abolished the anti-hyperalgesic effect of oxytocin [109]. An anti-nociceptive role of oxytocin was also proposed by Mou and colleagues [110]. They performed intrathecal administration of oxytocin in a rat model of bone cancer. Mechanical allodynia seemed to be attenuated in a dose-dependent manner after oxytocin administration, associated with an increase in the thermal heat threshold. Furthermore, repeated administration of oxytocin for 21 days prevented the upregulation of TLR-4 in the spinal cord [110]. The TLR-4 pathway is strongly implicated in cancer pain hypersensitivity [135] and its attenuation could provide pain relief for cancer patients [136]. In addition, an increase in the levels of pro-inflammatory cytokines in the spinal cord, such as TNF-α and IL-1β, was also observed in such rat model; interestingly, oxytocin treatment was able to prevent such an increase. This study suggests that oxytocin might ameliorate cancer pain by modulating the expression of TLR-4 and pro-inflammatory cytokines in the spinal cord [110]. The anti-inflammatory effect of oxytocin administration in a rat model of carrageenan-induced inflammation has also been evaluated [43]. Indeed, carrageenan injection induced an inflammatory pain state, resulting in an increase of spinal levels of inflammatory markers such as TNF-α, IL-1β, and macrophage inflammatory protein-1α. Furthermore, local edema, hyperalgesia, and mechanical allodynia occurred [137]. In this animal model, it was demonstrated that subcutaneous administration of oxytocin reduced local edema and neutrophil infiltration induced by carrageenan injection into the paw. Additionally, oxytocin raised the pain threshold for thermal and mechanical stimuli from 1 h to 6 h after injection [43]. Yu et al. instead revealed that this lag time (from 1 h to 6 h made by subcutaneous injection) appeared to be reduced by intrathecal injection of oxytocin, in an equivalent rat model of carrageenan-induced inflammation. Indeed, the peak of anti-nociceptive action for both mechanical and thermal stimulation resulted 5 min after oxytocin administration. Interestingly, the administration of the oxytocin receptor antagonist atosiban was able to increase the hypersensitivity in rats with inflammation when administered alone and to attenuate the anti-hyperalgesic effects of the exogenous oxytocin when administered centrally before oxytocin. Moreover, the authors suggested a possible involvement of the opioid system in the anti-nociceptive action of oxytocin. Indeed, its effect was first attenuated by administration of a non-selective opioid receptor antagonist (Naloxone) and then by two selective opioid receptor antagonists (targeting either the mu or the kappa opioid receptors) but not by a delta-receptor selective antagonist, excluding a possible action through these receptors [111]. It seems that the central oxytocinergic system is susceptible to inflammatory stimuli since the first step of life. Thus, Lee and colleagues demonstrated that inflammatory pain induced by subcutaneous formalin injection in neonatal rats induces a marked reduction in the expression of the oxytocin receptor in the hippocampus and cortex. This highlights a dysregulation of the oxytocinergic system at the CNS level in a condition of neonatal inflammatory pain, which could lead to the development of neurodevelopmental diseases at a young age [97]. The evidence collected in this paragraph is summarized in Table 1.

### 3.3. Obesity and Diabetes

Obesity is a polygenic and multifactorial condition that represents a very concerning public health issue affecting both developing and developed countries [138,139].

The onset of obesity is often associated with the development of several chronic complications including type 2 diabetes (T2DM) and elevated hypertriglyceridemia, dyslipidemia, and hypertension [140]. Both obesity and T2DM are also associated with the activation of the immune system [141]. Indeed, a well-described feature of obesity and T2DM is chronic, unresolved tissue inflammation that differs from the classical inflammatory response since it is characterized by a chronic, low-intensity reaction [142]. Particularly, excessive calorie intake and increased fat accumulation trigger the production of effector molecules such as cytokines [143]. This production leads to the chronic, low-grade inflammatory status that induces, in the metabolic tissues, the recruitment and activation of many mature immune cells (including monocytes that differentiate into macrophages and express the classical proinflammatory phenotype) and of other cells, such as adipocytes, that modify the tissue environment [143,144]. Moreover, it has been shown that the activation of effector molecules of inflammation, such as TNF-α, contributes to desensitizing the insulin signaling pathways [143]. Many studies in rodents and some in humans have shown that the oxytocinergic system can affect the regulation of body weight and metabolism [27,90,145,146,147,148,149]. Thus, oxytocin has recently gained attention for its anti-inflammatory properties as a putative treatment for obesity as well as for glucose- and insulin-related disorders [150,151,152,153]. Different studies support this hypothesis, demonstrating that adipocytes and macrophages express oxytocin receptors [154,155]. In particular, Yi et al. demonstrated that both adipocyte differentiation and fat accumulation induce oxytocin receptor up-regulation in mice [154]. Moreover, Szeto et al. demonstrated an upregulation of the oxytocin receptor in human macrophages in response to an inflammatory stimulus, mediated via NF-κB pathway activation [155]. These studies strongly suggest that the adipose tissue might represent an important target for the anti-inflammatory actions of oxytocin. Therefore, Nation et al. investigated the effect of oxytocin on adipose tissue inflammation. To this aim, they used an animal model with impaired ability to clear cholesterol from the circulation, due to the lack of the lipid carrier protein, apolipoprotein E (apoE^−/−^ mice); this resulted in elevated plasma cholesterol levels and development of extensive atherosclerosis and tissue inflammation. The exogenous oxytocin infusion significantly reduced the secretion of the pro-inflammatory cytokine IL-6 at the level of adipose tissue (epididymal fat) of apoE^−/−^ mice compared with vehicle control animals [112]. Altirriba et al. aimed to determine whether a chronic oxytocin treatment could be beneficial for obesity and its comorbidities. In addition to body weight and glucose metabolism assessment, adipose tissue inflammation was measured by quantifying the number of macrophages in the epididymal fat depot. The leptin-deficient animal model (ob/ob mice) was selected for the study because it presents a more severe phenotype than diet-induced obese (DIO) mice and rats, with more extreme obesity and inflammation as well as higher basal glycemia and insulinemia; the authors demonstrated that the peripheral administration of oxytocin reduced the infiltration of adipose tissue by macrophages in ob/ob mice and such effect was accompanied by decreased body weight gain, thus suggesting a potential beneficial role of oxytocin in the treatment of alterations typical of obesity [113]. On the same line, Szeto et al. evaluated the effects of chronic oxytocin infusions on adipose tissue inflammation in the leptin receptor-deficient mice (db/db mice), which also represent a preclinical model of obesity, adipose tissue inflammation, and diabetes. The authors demonstrated that the peripheral oxytocin infusion reduced adipocyte size, macrophage infiltration, and IL-6 and TNFα mRNA expression and increased the expression of the anti-inflammatory adipokine, adiponectin. In such a model, the beneficial effect of oxytocin was also observed at the level of circulating plasma, where oxytocin reduced the level of marker of systemic inflammation, such as plasma serum amyloid A and increased circulating adiponectin (an anti-inflammatory plasma marker). Interestingly, the anti-inflammatory effect evoked by oxytocin was observed in the absence of weight loss or changes in glycemic control, suggesting that oxytocin works directly to suppress inflammation of adipose tissue rather than through the reduction in adipose tissue mass or glycemic regulation [114].

Garrido-Urbani et al. assessed the anti-inflammatory effect of oxytocin both in in vitro and in vivo studies. For in vitro studies, bone marrow-derived macrophages were differentiated into inflammatory and resting phenotypes; the stimulation with oxytocin decreased pro-inflammatory macrophages’ differentiation without affecting the resting macrophages’ population, thereby leading to an anti-inflammatory phenotype (decreased macrophages’ proinflammatory/resting ratio). Moreover, it was observed that oxytocin, at the level of pro-inflammatory macrophages, dose-dependently decreased TNF-α secretion. Instead, for in vivo studies, DIO and lean mice were peripherally treated with oxytocin for 2 weeks, and eating behavior, body weight, and local adipose tissue inflammation were evaluated. The results showed no change in macrophages’ proinflammatory/resting ratio, whereas the decreased TNF-α expression observed already in vitro was confirmed and was accompanied by decreased body weight and an improvement in glucose tolerance in DIO mice [115]. Finally, only one clinical study, by Akour et al., aimed to evaluate the differences in oxytocin plasma levels and their potential association with inflammatory and anti-inflammatory markers in diabetic (MetS-T2DM), prediabetic (MetS-prediabetics), and non-diabetic with metabolic syndrome (MetS-only) patients. The results showed that oxytocin plasma levels were significantly lower in MetS-T2DM and MetS-prediabetics patients compared with MetS-only subjects. Moreover, plasma oxytocin correlated differently with inflammatory and anti-inflammatory markers in the entire MetS groups of participants, with a positive correlation with the anti-inflammatory cytokines IL-10 and IL-6 and a negative correlation with the pro-inflammatory cytokine TNF-α [116]. The evidence collected in this paragraph is summarized in Table 1.

### 3.4. Gastrointestinal Inflammatory Diseases

Oxytocinergic signaling appears to play an important role in multiple GI functions that are under neuronal regulation. Like brain oxytocin, enteric oxytocin is restricted to enteric neurons; however, enteric oxytocin receptors are not exclusively neuronal [156]. Indeed, the oxytocin receptor is expressed by both the enteric neurons and the mucosal epithelium, especially in junctional complexes at crypt-villus boundaries [119]. Welch et al. tested the hypothesis that oxytocin, in combination with secretin (S, a best-known duodenal hormone), modulated the transmission of signals from the inflamed bowel to the brain. In fact, by using an animal model of colitis induced by rectal administration of 2,4,6-trinitrobenzene sulfonic acid (TNBS), the authors demonstrated that oxytocin and S in combination attenuated intestinal inflammation by decreasing inflammatory infiltrates into the colon and by affecting the colon expression of TNF-α and IFN-γ; moreover, oxytocin and S co-administration prevented the transmission of inflammation-evoked signals to paraventricular nucleus, amygdala, and piriform cortex [117]. Subsequently, Welch et al. demonstrated in OXYR-KO mice that oxytocin/oxytocin receptor signaling plays an important role in multiple GI functions such as motility; it also decreases mucosal activation of enteric neurons, promotes enteric neuronal development and/or survival, and regulates proliferation of crypt cells and mucosal permeability. Moreover, in OXYR-KO mice in which colitis was induced by TNBS and dextran sulfate sodium, the activation of the local oxytocinergic signaling mitigated intestinal inflammation [37]. Klein et al., instead, aimed to evaluate the impact of colostrum oxytocin on markers of inflammation in isolated newborn rat gut villi, and suggested that colostrum oxytocin is capable of dampening inflammation on postnatal gut villi and induced autophagy [118].

Furthermore, Margolis et al. tested the idea that oxytocin might be able to counteract the proinflammatory drive that occurs in the bowel in the necrotizing enterocolitis (NEC), a severe intestinal inflammatory disease that occurs in 10–15% of premature infants. In a NEC animal model, they found that exogenous oxytocin was able to decrease transcription of proinflammatory chemokines and cytokines and enhanced transcription of anti-inflammatory gene products. Moreover, the administration of the oxytocin receptor antagonist atosiban exacerbated the NEC, supporting the potential beneficial effect of oxytocin in such condition [119].

Finally, in a recent work, Tamer et al. investigated the impact of regular exercise on oxidative gastric injury and the role of oxytocin receptor activity in the anxiolytic and anti-inflammatory actions of exercise. Adult rats, at the end of an exercise/sedentary protocol, were treated with atosiban and then, to induce ulcer, acetic acid was applied onto the gastric serosa. The authors demonstrated that the inhibition of oxytocin receptors by atosiban exacerbated the expression of pro-inflammatory cytokines in the stomachs with ulcers of exercised rats. Moreover, when the rats regularly exercised before ulcer induction, a restored oxytocinergic signaling at both hypothalamic and myenteric levels was observed compared with sedentary rats with gastric ulcers. Thus, these findings suggest that exercise reverted the down-regulated expression of hypothalamic oxytocinergic neurons as well as gastric oxytocin receptors; moreover, the gastroprotective effects of exercise are partially reversed by the inhibition of oxytocin receptors [120]. The evidence collected in this paragraph is summarized in Table 1.

### 3.5. Cardiovascular Diseases

Inflammation has definitely been established as central to the development and complications of several cardiovascular disease (CVD) [157,158,159]. Indeed, elevated markers of inflammation, such as C-reactive protein (CRP) and serum amyloid A, have been supposed to be predictive of future CVD [160,161,162]. Therefore, rising evidence supports the hypothesis that targeting the innate immune system and specific inflammatory proteins and/or pathways may be effective in reducing the risk of cardiovascular events [163]. Interestingly, oxytocin and oxytocin receptors are expressed within vascular and cardiac tissue both in rodents and humans and play a key role in the regulation of cardiovascular homeostasis [112,121]. Indeed, oxytocin regulates blood volume, heart contractility, vascular tone, and vascular regrowth and remodeling [112,121]. Despite an increasing number of studies supporting the homeostatic regulation of oxytocin in the cardiovascular system [164], little is known about its role in the injured heart. Therefore, to better understand the potential role of oxytocin in cardiovascular pathology, different in vitro and in vivo studies have emerged in the past years, among which a variety of those support the interplay among oxytocin signaling, inflammation, and CVD. For instance, in vitro studies performed in endothelial cells showed that oxytocin attenuates pathophysiological processes involved in atherosclerotic lesion formation by dampening IL-6 secretion, thus suggesting a possible anti-inflammatory effect in this context [121].

Moreover, the anti-inflammatory effects of oxytocin treatment have been shown also in a rat model of myocardial infarction, where oxytocin reduced cell infiltration, such as neutrophils, macrophages, and T-lymphocytes in infarcted cardiac areas; interestingly, such effect was accompanied by a reduction in the expression of proinflammatory cytokines, such as TNF-α, IL-6, and IL-1β, and stimulation of the anti-inflammatory cytokine TGF-β [122]. Moreover, in ApoE^−/−^ knockout mice, an animal model of hyperlipidemia and atherosclerosis, it was shown that chronic oxytocin treatment attenuated aortic atherosclerosis in a site-specific manner. Indeed, oxytocin-treated animals displayed in the thoracic aorta significantly less atherosclerosis compared with vehicle-treated animals and this effect was accompanied by a reduction of adipose tissue inflammation [112].

This result was supported by another study conducted in an animal model of dyslipidemia and atherosclerosis, the Watanabe Heritable Hyperlipidemic rabbit. In such a model, it was shown that chronic oxytocin treatment reduced not only atherosclerosis in the thoracic aorta but also the systemic inflammation. In particular, oxytocin-treated rabbits exhibited significantly lower levels of plasma CRP, lower expression of the proinflammatory adipokine IL-6, and higher expression of anti-inflammatory adipokine, adiponectin, if compared to vehicle-treated animals [123]. Moreover, a study performed in a rat model of pulmonary hypertension, characterized by right ventricular (RV) hypertrophy and inflammation, showed a downregulation of oxytocin receptor within the RV, which is associated with an increase of mRNA expression of the pro-inflammatory cytokines IL-1β and IL-6 [165]. Cardiac abnormalities are also a feature of obesity and diabetes, and, on this line, Plante et al. evaluated whether chronic oxytocin treatment prevented cardiac abnormalities associated with obesity and diabetes by using the db/db mice model. Interestingly, this animal model produced, in addition to a state of obesity and diabetes, also a deficiency in the cardiac oxytocin/ natriuretic system and developed systolic and diastolic dysfunction resulting from cardiomyocyte hypertrophy, fibrosis, and apoptosis. The authors demonstrated that oxytocin treatment in db/db mice reduced the activity of NF-κB and the mRNA expression of the inflammatory cytokines IL-1β and IL-6, which resulted elevated in the cardiac tissue of db/db vehicle-treated mice; moreover, oxytocin treatment resulted in a decrease of the left ventricular weight and collagen volume fraction, thus improving cardiac function [124]. Subsequently, two studies, from the same research group, were conducted to evaluate the effect of the central hypothalamic oxytocinergic activation in rat models of heart failure (HF). HF is characterized by an autonomic imbalance consisting of increased sympathetic activity and decreased parasympathetic tone. Therefore, with the aim of elevating the parasympathetic tone, hypothalamic oxytocin neurons were chronically selectively activated; to achieve this selective activation, Designer Receptors Specifically Activated by Designer Drugs were expressed in PVN oxytocin neurons and activated by injections of clozapine-N-oxide that increases the firing of those neurons for at least 1 h. It was demonstrated that this activation not only improved autonomic tone and cardiac function, but also reduced fibrosis and cardiac inflammation, particularly the expression of the pro-inflammatory cytokine IL-1β [125,126]. Additionally, in the doxorubicin-induced cardiomyopathy rat model, oxytocin treatment reduced oxidative, apoptotic, and inflammatory activity and provided better tissue integrity [127]. Finally, Xiong and colleagues demonstrated in a myocardial ischemia/reperfusion (I/R) injury rat model that oxytocin inhibited the degranulation of cardiac mast cells induced by the I/R injury and downregulated the expression of inflammatory factors such as High Mobility Group Box-1 and NF-κB [128]. Overall, the results reported highlight that the oxytocinergic system, besides having a key role in the homeostatic regulation of cardiac functions, may constitute an important target for the prevention and possibly for the treatment of inflammation associated with CVD. The evidence collected in this paragraph is summarized in Table 1.

### 3.6. COVID-19

Due to the particular historical period we are experiencing, and based on the emerging literature, as a final stage of this review we also added information about the possible involvement of the oxytocinergic system in inflammation induced by coronavirus disease (COVID)-19. The pandemic spread of the SARS-CoV-2 virus has led to the knowledge of a new disease, COVID-19, resulting from the infection of the virus. The main event that characterizes this disease is hyperinflammation or the onset of a cytokine storm that leads in severe cases to the development of acute distress syndrome. Immune overreaction is often not limited to just the lungs, but it can become systemic, causing hypercoagulation and multiple-organ failure [166,167,168]. The entry of the virus into cells appears to be mediated mainly by the angiotensin-converting enzyme 2 receptor, also using a co-receptor called dipeptidyl-peptidase (DPP) 4/CD26. Therefore, DPP4 was considered as a possible target to limit the entry of the virus into cells and, therefore, prevent the massive activation of the immune system and consequent hyper-inflammation using its inhibitors such as gliptins [169]. Chittepu et al. proposed oxytocin as a natural inhibitor of DPP4, demonstrating its inhibitory potential on the biological activity of DPP4 through molecular docking studies [170]. Based on this evidence, Diep et al. advanced the hypothesis that oxytocin may be a direct viral inhibitor against SARS-CoV-2 [171]. Anti-inflammatory and pro-immune properties have been associated with oxytocin through studies on carbetocin, its structural analogue and peripheral agonist of the oxytocin receptor. The anti-inflammatory action exerted by oxytocin and the sustenance of the immune response seem to be mediated by the inhibition of the signaling of the transcription factor NF-kB [172]. The therapeutic potential of oxytocin in the treatment of COVID-19 and/or in reducing the hospitalization period is also supported by Buemann et al., who reported the capability of oxytocin in dampening the inflammatory response driven by neutrophils in different animal models and its potential in decreasing the infiltration of inflammatory tissue cells [41].

Although further studies are needed to investigate the anti-inflammatory potential of oxytocin in patients with COVID-19, these studies open the way to further investigating the role of the oxytocinergic system as a valid therapeutic target for the prevention and/or mitigation of the cytokine storm triggered by SARS-CoV-2 infection.

## 4. Conclusions

Inflammation is primarily a physiological and beneficial process; however, excessive and non-resolving inflammatory responses can cause or contribute to tissue damage and the pathogenesis and progression of many human diseases including neuropsychiatric, metabolic, and cardiovascular pathologies.

The epidemiological spread of diseases showing an inflammatory component is pushing the interest of the scientific community in further studying the inflammatory machinery to deeply understand the molecular and cellular mechanisms underpinning the inflammatory response, to open the way for novel pharmacological interventions.

Oxytocin is a natural and well-known neuropeptide involved in several pathophysiological mechanisms throughout the body. The evidence reviewed in this work highlights the significant role of oxytocin in cellular and molecular pathways shared by both central and systemic inflammation. In particular, such report supports a beneficial effect of the stimulation of both the central and peripheral oxytocinergic system to control inflammation occurring in different diseases (Table 1). Finally, although very few studies are reported in the literature, we also added evidence that oxytocin might be of interest as a targeting system for the treatment of COVID-19.

It can be concluded that the central oxytocinergic system is possibly one of the mechanisms that participate in the coordination of different aspects of the inflammatory process. This concept should promote future studies aimed at elucidating the mechanistic details underlying the regulation of oxytocin secretion during inflammatory challenges and the coordinated processes between oxytocin and cytokines in the different organs affected.

Finally, the evidence collected in this review strongly supports the idea that the oxytocinergic system might represent a new pharmacological target for the treatment of inflammation in selected chronic diseases.

## Figures and Tables

**Table 1:** Role of the oxytocinergic system in inflammation occurring in different chronic diseases.

***Depression***				
**Subjects**	**Targets**	**Analysis**	**Effect**	**Reference**
Mice model of maternal separation	Hippocampus	i.c.v. oxytocin administration	Attenuated depressive-like behavior, Restored GSH, decreased ROS Decreased TNF-α, IL-1β, and TLR-4	[63]
Mice subjected chemotherapy	Hippocampus	i.c.v. oxytocin administration	Attenuated depressive-like behavior Decreased IL-6	[88]
Adolescent rats from postpartum depressive dams	Hippocampus	i.p. oxytocin administration	Increased social investigation, stimulated neurogenesis Decreased neuroinflammation (IL-6:IL-10 ratio)	[89]
***ASD***				
**Subjects**	**Targets**	**Analysis**	**Effect**	**Reference**
Autistic male patients	Plasma	Endogenous oxytocin levels	Negative correlation between oxytocin and IFN-γ-induced protein-16	[101]
Autistic mice from valproate treated dams	Hippocampus Amygdala	i.n. oxytocin administration	Improved anxiety-, depressive-like, and social behaviors, Decreased IL-6, IL-1β, TNF-α Decreased ROS, restored GSH	[102]
	Hippocampus Cortex		Decreased Iba1	
OXYR-KO mice	Brain	Endogenous oxytocin levels	Decreased PSD95, increased Iba1	[70]
***Inflammatory Pain***				
**Subjects**	**Targets**	**Analysis**	**Effect**	**Reference**
Rat model of peripheral painful inflammatory sensitization	Spinal cord	Optogenetic stimulation of oxytocin parvocellular neurons	Attenuated nociceptive transmission	[108]
Rat model of peripheral painful inflammatory sensitization	Spinal cord	i.p. administration of selective oxytocin receptor agonist LIT-001	Increased mechanical and thermal pain threshold	[109]
Rat model of bone cancer	Spinal cord	i.t. oxytocin administration	Increased mechanical and thermal pain thresholds Decreased IL-1β, TNF-α, and TLR-4	[110]
Rat model of carrageenan-induced inflammation	Spinal cord	s.c. oxytocin administration	Increased mechanical and thermal pain thresholds	[43]
	At the injection site of carrageenan (hind paw)		Decreased local edema and neutrophil infiltration	
Rat model of carrageenan-induced inflammation	Spinal cord	i.t. oxytocin administration	Increased mechanical and thermal pain thresholds	[111]
***Obesity and Diabetes***				
**Subjects**	**Targets**	**Analysis**	**Effect**	**Reference**
apoE^−/−^ mice	Adipose tissue	s.c. oxytocin infusion	Decreased IL-6	[112]
ob/ob mice	Adipose tissue	s.c. oxytocin infusion	Decreased body weight gain	[113]
			Decreased macrophages infiltration	
db/db mice	Adipose tissue	s.c. oxytocin infusion	Decreased adipocyte size Decreased macrophages’ infiltration, IL-6, and TNF-α Increased adiponectin	[114]
	Plasma		Decreased plasma serum amyloid A	
Mice C57bl/6JRj	Bone marrow-derived macrophages	Stimulation with oxytocin	Decreased TNF-α secretion	[115]
DIO mice		s.c. oxytocin infusion	Decreased body weight and improved glucose tolerance	
	Macrophages		Decreased TNF-α secretion	
Human MetS subject	Plasma	Endogenous oxytocin levels	Decreased TNF-α Increased IL-10 and IL-6	[116]
***Gastrointestinal Tract Pathologies***				
**Subjects**	**Targets**	**Analysis**	**Effect**	**Reference**
Rat model of colitis	Colon	i.v. oxytocin + secretin administration	Prevented transmission of inflammation-evoked signals to PVN, AMY, and piriform cortex Decreased inflammatory infiltration Decreased TNF-α and INF-γ	[117]
Model of colitis induced in OXYR-KO mice	Intestine	s.c. oxytocin administration	Decreased levels of TNF- α and CCR5	[37]
Isolated newborn rat gut villi	rat gut villi	Oxytocin colostrum level	Increased the inhibition of NF-kB pathway	[118]
Murine NEC model	Intestine	Peripheral oxytocin administration	Decreased transcription of proinflammatory chemokines and cytokines Increased transcription of anti-inflammatory gene products	[119]
Rat model of gastric ulcer	stomach	i.p. administration of oxytocin receptor antagonist atosiban	Increased pro-inflammatory cytokine expressions	[120]
***Cardiovascular Diseases***				
**Subjects**	**Targets**	**Analysis**	**Effect**	**Reference**
Human cells	Primary aortic endothelial cells	Stimulation with oxytocin	Attenuated atherosclerotic lesion formation Decreased IL-6	[121]
Rat model of myocardial infarction	Infarcted cardiac areas	s.c. oxytocin infusion	Decreased macrophages’, neutrophils’, and Lymphocytes’ infiltration Increased TGF-β Decreased IL-6, IL-1β, TNF-α	[122]
apoE^−/−^ mice	Aorta	s.c. oxytocin infusion	Decreased atherosclerosis	[112]
Watanabe heritable hyperlipidemic rabbit	Aorta	s.c. oxytocin infusion	Decreased atherosclerosis	[123]
	Plasma		Decreased CRP	
	Adipose tissue		Decreased IL-6 Increased adiponectin	
db/db mice	Cardiac tissue	s.c. oxytocin infusion	Decreased left ventricular weight and collagen volume fraction Decreased IL-6, IL-1β, NF-κB	[124]
Rat model of heart failure	Cardiac tissue	Selective activation of PVN oxytocin neurons	Improved cardiac function Decreased fibrosis Decreased IL-1β	[125,126]
Rat model of cardiomyopathy	Myocardial tissue	i.p. oxytocin administration	Improved tissue integrity Decreased oxidative stress, apoptosis, and inflammation	[127]
Rat model of I/R	Cardiac tissue	i.p. oxytocin administration	Inhibited cardiac mast cells’ degranulation Decreased NF-κB and High Mobility Group Box-1	[128]

Amygdala (AMY); autism spectrum disorders (ASD); C-C chemokine receptor (CCR5); C-reactive protein (CRP); glutathione (GSH); ionized calcium-binding adapter molecule (Iba); intracerebroventricular (i.c.v); interferon (IFN); interleukin (IL); intranasal (i.n.); intraperitoneal (i.p.); intrathecal (i.t.); intravenous (i.v.); ischemia/reperfusion (I/R); metabolic syndrome (MetS); nuclear factor (NF); oxytocin-receptor knockout (OXYR-KO); postsynaptic density protein (PSD); paraventricular nucleus (PVN); reactive oxygen species (ROS); subcutaneous (s.c.); transforming growth factor (TGF); toll-like receptor (TLR); tumor necrosis factor (TNF).

## Abbreviations

CNS	central nervous system
PLC	phospholipase C
Ca^2+^	calcium
LPS	lipopolysaccharide
ROS	Reactive oxygen species
GSH	glutathione
NPC	neural progenitor cells
MAPK	mitogen-activated protein kinase
ERK	extracellular signal-regulated kinase
GFAP	glial fibrillar acidic protein
CD	cluster of differentiation
CNPase	2′,3′-Cyclic-nucleotide 3′-phosphodiesterase
TGF	transforming growth factor
AQP	aquaporin
TNF	tumor necrosis factor
IL	interleukin
COX	cyclooxygenase
iNOS	inducible nitric oxide synthase
Iba	ionized calcium-binding adapter molecule
TLR	toll-like receptor
NF	nuclear factor
BDNF	brain-derived neurotrophic factor
IFN	interferon
PSD	postsynaptic density protein
CRP	C-reactive protein
CFA	complete Freund adjuvant
DPP	dipeptidyl-peptidase
ASD	autism spectrum disorder
T2DM	type 2 diabetes mellitus
DIO	diet-induced obesity
GI	gastrointestinal
IBD	inflammatory bowel diseases
OXYR-KO	oxytocin receptor knockout
TNBS	2,4,6-trinitrobenzene sulfonic acid
NEC	necrotizing enterocolitis
CVD	cardiovascular disease
HF	heart failure
I/R	ischemia/reperfusion
COVID	coronavirus disease
